# Unravelling the underpinnings of hyperprogression and immunotherapy: back to the bench

**DOI:** 10.18632/oncotarget.28098

**Published:** 2022-01-05

**Authors:** Vivek Subbiah, Razelle Kurzrock

**Keywords:** hyperprogression, immunotherapy, mouse model, PD-1, checkpoint

## INTRODUCTION

The advent of checkpoint immunotherapies has altered the landscape of multiple cancers, and a large number of patients benefit from long lasting durable responses. However, there are a minor subset of patients who are unresponsive to immunotherapy and, even worse, some exhibit dramatic progression at an accelerated pace that would be unexpected with the trajectory of their natural history of disease [1–3]. This provocative phenomonen has been a hot topic in oncology and is termed “hyperprogressive disease” [2, 4]. This is in addition to another unusual pattern of response known as “pseudoprogression,” wherein tumors initially progress and then start responding to therapy. While there is universal acknowledgement of pseudoprogression, oncologists are polarized with this “hyperprogression” as a response to immunotherapy. In addition, there have been varied clinical definitions that define hyperprogressive disease (HPD), though commonly this is considered a tumor growth rate (TGR) that was at least twofold greater during immunotherapy than immediately before immunotherapy with a >50% increase in tumor burden and a <2-month ‘time to treatment failure’ [1–4]. Despite varied clinical definitions, the underlying theme is that there is dramatic acceleration of disease and multiple groups have independently reported and confirmed HPD clinically with incidence ranging from 4–29% reported retrospective studies [2].

There is a growing body of evidence on the science behind this phenomenon. Lo Russo et al. in a translational study showed the role of innate immunity in mediating HPD via Fc/FcR triggering on macrophages by anti–PD-1 antibody [5]. In their study they showed that all patients with HPD showed tumor infiltration by M2-like CD163+CD33+PD-L1+ clustered epithelioid macrophages [5]. Kato et al. showed patients with *MDM2* amplification or *EGFR* mutations exhibited an increased rate of hyperprogression to single agent immunotherapy [4]. Xiong et al. reported the genomic and immunologic landscapes between pre-treatment and post-treatment samples of two patients whose tumors showed HPD after immune checkpoint inhibitor treatment [6]. Somatic mutations of *TSC2* and *VHL* (tumor suppressor genes) in addition to transcriptional upregulation of IGF-1, ERK/MAPK, PI3K/AKT, and TGF-β oncogenic pathways were seen in post-treatment HPD specimens versus pre-treatment samples [6]. Immunologically, HPD tumors demonstrated an increase in the ILC3 subset of the innate lymphocyte system post- immunotherapy [6]. Kamada et al. [7] generated mice with T-cell-specific PD-1 deficiency, and investigated the role of PD-1 in murine T-reg cells, indicating that blockade promotes cell cycling of T-reg cells and augments T-reg cell-mediated immune suppression [7]. Despite several studies the underpinnings and basic mechanisms of this conundrum are unclear and there is an urgent need to develop pre-clinical experimental models to better understand the biology behind this phenomenon.

**Figure 1:**
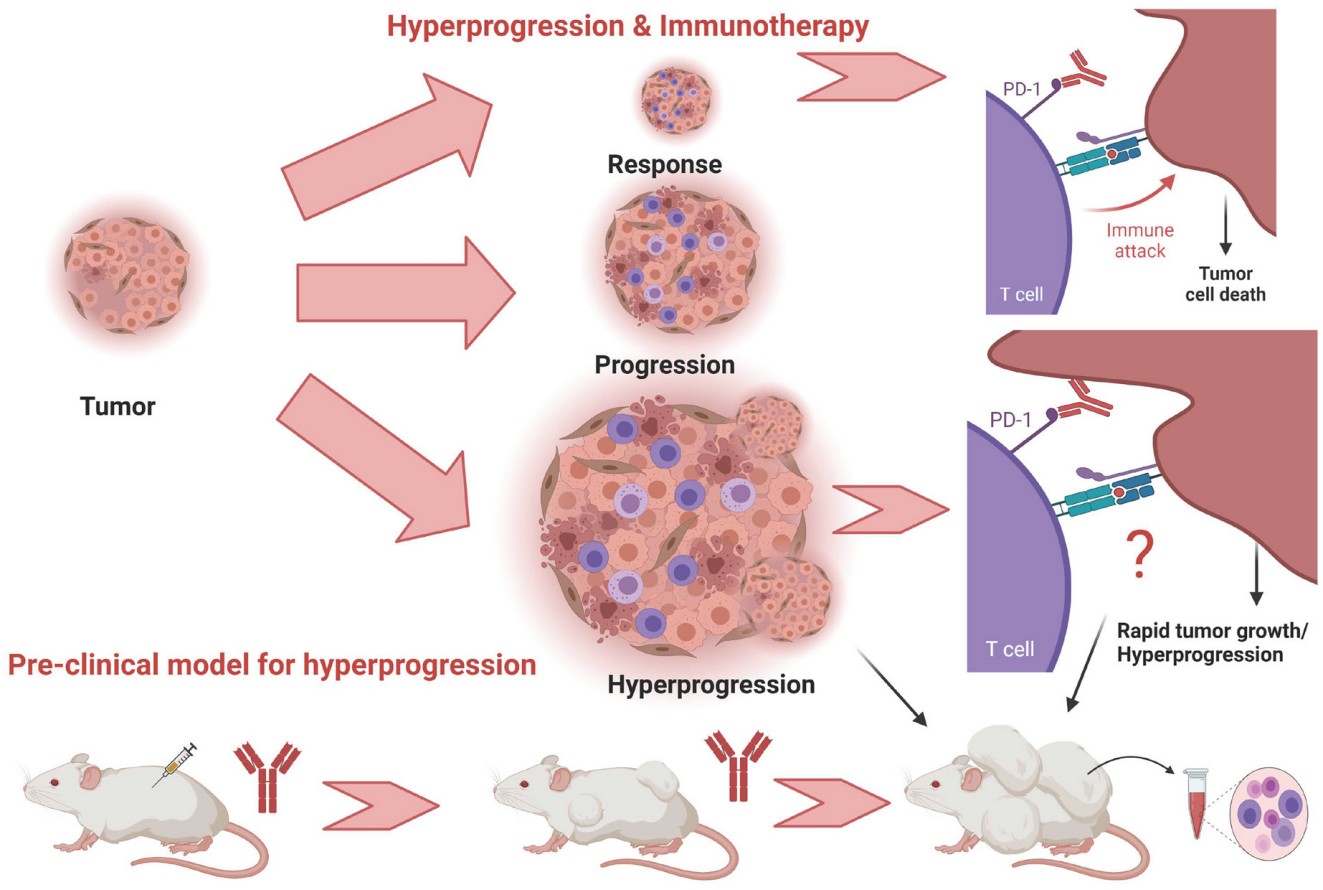
Hyperprogression and immunotherapy. Patterns of response to immunotherapy include response, progression and hyperprogression. Pre-clinical models to elucidate hyperprogression are crucial in understanding the biology of hyperprogression. Created with https://biorender.com.

With this background, the paper by Sahin et al. assumes significance [8] as they present a humanized mouse model recapitulating HPD that may aid in gaining more insights into the underlying biology of HPD (Figure 1). In their elegant set of experiments, using athymic mice, they generated xenograft models of subcutaneous human colorectal tumors of cell lines HCT-116 p53−/−, HCT-116 p53+/+, and DLD-1 (mutant p53/Ser241Phe). This was a model bearing mismatch repair-deficient (MMR-d) human colon carcinoma HCT116 p53-null (but not wild-type p53) tumor xenograft. After establishing this, they conducted intraperitoneal injection of TALL-104, CD8+ human Cytotoxic T cells (CTLs), before treating with anti-PD1 pembrolizumab immmunotherapy intraperitoneal injection and determined the effects of the therapy on tumor growth. They observed rapid tumor growth post anti-PD1 therapy and showed that this model was MDM2 or MDM4/MDMX independent. Moreover they performed comprehensive human cytokine profiling in this model as well.

It is crucial that this HPD model has a functioning human T-cell line, TALL-104, which shows phenotypic characteristics of cytotoxic T lymphocytes. Previous murine models reported in literature in this context did not have human mature functioning T-cells. It is important to note that the most commonly used immune checkpoint inhibitors, pembrolizumab and nivolumab are human IgG4k monoclonal antibodies against PD-1 and do not recognize murine PD-1. Because of this issue, for pre-clinical experiments they are substituted with anti-mouse PD-1 antibodies. Therefore humanized mouse models as established here are critical and may reflect biology of human tumors more closely and may be better for evaluation in pre-clinical studies. Intriguingly, soluble cytokines play a role in both tumor response or tumor growth and elucidation of cytokine profiles may inform regulatory role in the tumor micro-environment. Several cytokines including IFN-γ, TRAIL R2/TNFRSF10B, TRANCE/TNFSF11/RANK L, CCL2/JE/MCP-1, Chitinase 3-like 1, IL-4 and TNF-α were seen in the human cytokine profiling samples with a unique response in the HPD model that may uncover potential biomarkers, explain potential immune mechanisms, in addition to providing clues to therapy strategies. The need and evolution of better and better robust pre-clinical models always exists, as they are not human. Nevertheless, there is no denying that pre-clinical models have aided human research, advanced translational research and impacted human lives. Understanding unusual patterns of response and/ or resistance to immunotherapies may provide key knowledge to develop therapies in patients who develop HPD. This pre-clinical model is one such step in our efforts to unravel the underpinnings of HPD.
